# Optimisation of indole acetic acid production by *Neopestalotiopsis aotearoa* endophyte isolated from *Thymus vulgaris* and its impact on seed germination of *Ocimum basilicum*

**DOI:** 10.1186/s12896-024-00872-3

**Published:** 2024-07-06

**Authors:** Sayeda A. Abdelhamid, Mostafa M. Abo Elsoud, A. F. El-Baz, Ashraf M. Nofal, Heba Y. El-Banna

**Affiliations:** 1 Department of Microbial Biotechnology, National Research Centre, Cairo, Egypt; 2 Department of Industrial Biotechnology, GEBRI, University of Sadat City, Sadat City, Menofia, Egypt; 3 Department of Sustainable Development, Environmental Studies and Research Institute, University of Sadat City, Menofia, Egypt; 4 Department of Vegetable and Floriculture, Faculty of Agriculture, Mansoura University, Mansoura, Egypt

**Keywords:** Endophytic fungi, Medicinal plants, *Neopestalotiopsis aotearoa*, Indole acetic acid, Seed germination

## Abstract

**Background:**

Microbial growth during plant tissue culture is a common problem that causes significant losses in the plant micro-propagation system. Most of these endophytic microbes have the ability to propagate through horizontal and vertical transmission. On the one hand, these microbes provide a rich source of several beneficial metabolites.

**Results:**

The present study reports on the isolation of fungal species from different in vitro medicinal plants (i.e., *Breynia disticha major, Breynia disticha, Duranta plumieri, Thymus vulgaris, Salvia officinalis, Rosmarinus officinalis, and Ocimum basilicum* l) cultures. These species were tested for their indole acetic acid (IAA) production capability. The most effective species for IAA production was that isolated from *Thymus vulgaris* plant (11.16 µg/mL) followed by that isolated from sweet basil plant (8.78 µg/mL). On screening for maximum IAA productivity, medium, “MOS + tryptophan” was chosen that gave 18.02 μg/mL. The macroscopic, microscopic examination and the 18S rRNA sequence analysis indicated that the isolate that given code T4 was identified as *Neopestalotiopsis aotearoa* (T4)*.* The production of IAA by *N. aotearoa* was statistically modeled using the Box-Behnken design and optimized for maximum level, reaching 63.13 µg/mL. Also, IAA extract was administered to sweet basil seeds in vitro to determine its effect on plant growth traits. All concentrations of IAA extract boosted germination parameters as compared to controls, and 100 ppm of IAA extract exhibited a significant growth promotion effect for all seed germination measurements.

**Conclusions:**

The IAA produced from *N. aotearoa* (T4) demonstrated an essential role in the enhancement of sweet basil (*Ocimum basilicum*) growth, suggesting that it can be employed to promote the plant development while lowering the deleterious effect of using synthetic compounds in the environment.

**Supplementary Information:**

The online version contains supplementary material available at 10.1186/s12896-024-00872-3.

## Introduction

Preserving the ecological balance is one of the major global objectives. It is crucial to identify substitutes in agrosystems that boost output while reducing the need for artificial chemical products. The use of efficient biostimulants is one strategy that could be used to accomplish this goal. Endophytes, which are organisms that live in the tissues of healthy plants without harming the plants they inhabit [1, 2] may be a good fit in that purpose. Endophytic fungi shield plants against disease using a variety of strategies, including nutritional rivalry with pathogens, antibiotic production, and the activation of defence mechanisms [3] and moreover, they contain a wealth of bioactive substances that play various functions [4, 5]. Numerous fungal endophytic species have previously been documented to stimulate host growth [6] and provide their hosts with different advantages [7] such as it increases nitrogen fixation in the soil [8], phosphate solubilization [9], promote the synthesis of siderophores [10], and impact the synthesis of enzymes [11] For example, *Fusarium oxysporum, Cladosporium fulvum*, and *Phytophthora infestans* demonstrated antiparasitic action [9]. Additionally, endophytic plant-fungi interactions may potentially influence plant development by producing phytohormones [9] such as gibberellins and indole-3-acetic acid (IAA) [12, 13]. Numbers of studies have shown that certain endophytes produce phytohormones such as cytokinins, indole-3-acetic acid, and gibberellins to promote the growth of their host plants [14, 15].

Auxin, abscisic acid, and gibberellins are examples of plant hormones that control numerous physiological and metabolic functions of plants. In addition to plants, microorganisms also create these intriguing compounds [16, 17]. One of the major auxin in plants is indole-3-acetic acid, which affects several organogenesis developmental features [18]. IAA is one of the most significant phytohormones, as it plays essential roles in regulating a variety of functions, such as embryogenesis, cell cycling, the formation of vascular tissues, seed germination, seedling growth, and the formation of several plant parts, including the roots and leaves [18–21]. The impact of IAA on seedling development and germination was reported in *Asparagus sprengri* [22], *Vigna radiate* [23]**,** and *Gossypium hirsutum* [24]. It was noted that hormone application, hormone concentration, particular developmental phases, and metabolic activities are strongly correlated to each other [25]. As evidenced by a prior study, an ideal IAA concentration enhanced seed germination while a greater one prevented it [26]. The advancement of biotechnology has produced new possibilities for the synthesis of plant growth hormones utilizing fungi [27]. As a result, efforts are being made worldwide to investigate the possibility of creating fungi that produce IAA in order to support plant growth and safeguarding sustainable agriculture. Many phylogenetic evidences indicate the individual development of IAA production in plants, fungi, and bacteria [28]. This current study aims to produce IAA form *Neopestalotiopsis aotearoa*; as a first record, optimization production conditions for maximum IAA titer and evaluation of the effect of the produced IAA on the germination and growth of sweet basil seeds.

## Materials and methods

### Collection of plant material samples and sterilization

In order to maximize the chances of getting endophytic fungi, collection was done from different ornamental and medicinal plants (i.e., *V. major, B. disticha, D. plumieri, T. vulgaris, S. officinalis, R. officinalis, and O. basilicum*). All the individual host plant samples were collected from healthy, disease-free plants from nursery plantations and herbal gardens at Mansoura University, Mansoura, Egypt (31° 2′ 44.592'' N; 31° 21′ 12.96'' E) for the isolation of endophytic fungi. Plant samples (stems) were freshly transported to the tissue culture laboratory of the Vegetable and Floriculture Department, Faculty of Agriculture, Mansoura University. Each plant sample was thoroughly cleaned with tap water to get rid of the dust suspended in it. Plants were then separated and divided into little parts (nodal segments about 1–1.5 cm) by using a sterile scalpel. Surface sterilization was done for each plant as described in earlier studies, which involved using Tween 20 for 30 s, 70% ethanol, and sodium hypochlorite (NaOCl) at the ideal concentration and duration for each plant, followed by a rinse with sterile distilled water in order to get rid of the residues of the chemicals used within the technique of sterilization [29].

All sterilized explants were transferred under a laminar air flow chamber into jars containing 25 ml of MSM basal medium [30] plus 3% sucrose and solidified with 0.7% agar. MSM pH was adjusted to 5.75 before agar addition and autoclaved at 121°C, 1.1 kg/cm^2^ for 25 min. The seeded cultures were incubated in growth chambers at 25 ± 1°C under white fluorescent lights for 16 light/8h dark.

### Isolation of the endophytic fungi 

The seeded plant segments were observed daily for the growth of the endophytic fungi. The endophytic fungi that grown from the different plant segments were transferred into MOS medium and endophytic fungi obtained from each plant: *V. major* (T1), *B. disticha* (T2), *D. plumieri* (T3), *T. vulgaris* (T4), *S. officinalis,* (T5), *R. officinalis* (T6), and *O. basilicum* (T7). The species were sub-cultured monthly and preserved on PDA slants under cooling conditions.

### Screening for the most potent IAA-producing isolate

Seven endophytic fungal species coded: T1, T2, T3, T4, T5, T6 and T7 have been used for the current study. A single fungal species has been selected from each plant and given the same code. Potato dextrose broth (PDB) supplemented with tryptophan (Tryp) has been inoculated with the endophytes under investigation. The inoculated media were incubated at 30°C for 7 days under static conditions. Once incubation has ended, the microbial cells were removed from the media by centrifugation and cell-free medium was assayed for IAA production using Salkowski reagent according to Ehmann, [31].

### Screening for media testing with the most potential species

The most highly selected IAA-producing fungal species were evaluated for their IAA production using different growth media, including potato dextrose broth (PDB), PDB + tryptophan (Tryp), Czapex Dox (Czapex), Czapex + Typ, MOS medium Abo Elsoud et al. [32], and MOS + Tryp. Dehydrated Potato dextrose broth powdered medium (SRL Chemicals, India). Czapex Dox medium (g/l): 30, Sucrose; 2, sodium nitrate; 1, dipotassium hydrogen phosphate; 0.5, potassium chloride; 0.01, Magnesium sulfate; 0.5, ferrous sulfate [33]. MOS (g/l): 1.29, ammonium chloride; 0.93, tryptophan; 8.66, mannitol; 5.88, potassium nitrate; 4.41, D ( +) mannose; 1, KH_2_PO_4_; and 0.5, MgSO_4_.7H_2_O [32]. These media were inoculated with the most potent species and incubated at 30°C for 7 days under static conditions. At the end of incubation, the fungal cells were centrifuged, and cell-free medium was assayed for IAA production.

### Identification of the most promising species

The fungal species with the highest IAA production (T4) was selected for morphological and molecular methods.

### Macroscopic, microscopic identification

Fungal isolate T4 was grown on Potato Dextrose Agar (PDA)**.** Identification of selected fungal isolate was carried out by using the morphological characteristics as colony diameter, a color of conidia, extracellular exudates, pigmentation and the color of reverse mycelium. Microscopic features were examined as conidial heads, fruiting bodies, a degree of sporulation, and the homogeneity characteristics of conidiogenous cells were observed by optical light microscope (10 × 90) Olympus CH40 according to Ainsworth [34].

### Molecular identification

The molecular identification using 18S rDNA by Sigma Scientific Services Co. (https://www.sigmaeg-co.com/) and data submitted to the National Centre for Biotechnology Information (NCBI) and given an accession number.

### Statistical modeling and optimization of IAA production

Statistical modeling of IAA production by *Neopestalotiopsis aotearoa*. Seven factors have been considered for Box-Behnken: D ( +) Mannose, Mannitol, Tryptophan, Ammonium chloride, Potassium nitrate, Temperature and Initial pH. Design-Expert software (Stat-Ease Inc., Minneapolis, MN, USA, version 7.0.0) was used for the design and analysis of the model data (Table 1).

**Table 1:** Box-Behnken design summary of IAA production by *N. aotearoa*

Factor	Name	Units	Low actual	High actual
**A**	D ( +) Mannose	g/l	0	10
**B**	Mannitol	g/l	0	10
**C**	Tryptophan	g/l	0	6
**D**	Potassium nitrate	g/l	0	6
**E**	Ammonium chloride	g/l	0	6
**F**	Temperature	°C	25	40
**G**	Initial pH	Unit	5	8

### Data analysis and statistics

At the end of the experiment, a partial sum of squares analysis of variance (ANOVA) was accomplished for the evaluation of the model and model variables, where a *P*-value of < 0.05 was used as a criterion for the significance of the different model terms.

### Extraction of IAA

IAA in the culture medium was extracted using ethyl acetate at a ratio of 1:2, vigorously shaken, and afterward let to stand for ten minutes [35]. This procedure was performed four times, and IAA in the ethyl acetate layer was collected, pooled, and permitted to evaporate under reduced pressure to obtain IAA for further investigations.

### Germination experiment

#### Source of seeds and surface sterilization for germination

Seeds of sweet basil (*O. basilicum*) were obtained from Agricultural Research Center, Egypt. Before beginning an experiment and in order to prevent contamination, the seeds were surface sterilized for thirty seconds in 70% ethanol, then for ten minutes in 5% (v/v) sodium hypochlorite. Finally, they were cleaned with sterile distilled water [36].

### Experimental design and treatments

Twenty seeds of each treatment were cultivated on autoclaved medical cotton in 250-ml jars and moistened with 25 ml of distilled water (control) or IAA hormone solution in five different concentrations (25, 50, 75, 100 and 150 ppm). Seeds were incubated in a growth chamber at 25 ± 2°C with 16 h of light, followed by an 8 h. dark cycle. Five replicates of each treatment were used in a complete randomized block design.

### Data analysis and statistics

Data were subjected to analysis of variance using one-way analysis of variance (ANOVA) by using the COSTATC statistical package [37]. The means of all examined treatment results have been contrasted using Duncan’s multiple range test at *P* = 0.05. The means were presented with standard errors.

### Germination indices

Seed germination was observed every day for fourteen days, seed was considered germinated when the tip of the radicle had grown free of the seed coat [38]. Germination percentage (%), germination initial time (day), speed of germination (seed /day) and mean germination time (day), days to 50% germination, velocity of germination (%), and root length (cm) of germinated seeds were calculated as follows:

- ▪ Germination percentage = Germinated seeds/Total seeds*x*100 [39].
- ▪ Germination initial time = The number of days for the first germination [40].
- ▪ Speed of Germination = Σ(ni/di), Where “n” is the number of seeds that germinated on the day and “I” is the number of days counted from the beginning of germination [39].
- ▪ Mean Germination time = (Σni x di)/Σni [39].
- ▪ Days to 50% germination were calculated from the date of sowing to the date of 50% seeds germination by daily counting germinated seeds / Petri dish [41].
- ▪ Coefficient velocity of germination = (N/(Σni x di)) × 100 [42].

## Results and discussion

### Endophytic isolation

Seven fungal endophytes coded as T1, T2, T3, T4, T5, T6 and T7 have been isolated from seven different healthy, disease-free ornamental and medicinal plants (i.e. *V. major, B. disticha, D. plumieri, T.vulgaris, S. officinalis, R. officinalis, and O. basilicum*), respectively, from Mansoura University, Mansoura, Egypt. Endophytic microorganisms play a critical role in plant growth that lives together, mostly, in a symbiotic relationship. This relationship was demonstrated by Khalil et al. [43], who discovered a range of functional fungal relationships in medicinal plants and isolated 15 Ascomycota fungal endophytes from healthy leaves of *Ephedra pachyclada*, a medicinal plant. Additionally, eight medicinal plant samples that had been surface-sterilized allowed Ibrahim et al*.* [44] to successfully extract eighteen endophytic fungi from the fresh leaves. Similarly, the largest fungal endophytic diversity was found in* Thymus* spp. [45], *Chaetocladus monostachys* and *Salvia rosmarinus* plants [46].

### Screening for the most potent IAA-producing species

**Fig. 1:**
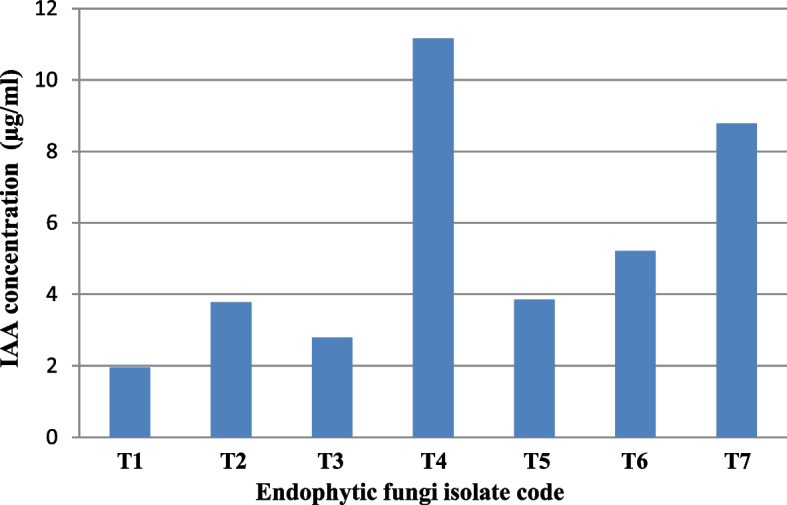
Screening for the most potent IAA-producing isolate on (PDB + Tryp) medium

Seven fungal species T1, T2, T3, T4, T5, T6 and T7 were isolated from different ornamental and medicinal plants *V. major*, *B. disticha*, *D. plumieri*, *T. vulgaris*, *S. officinalis, R. officinalis*, and *O. basilicum* on PDB + Tryp medium. The fungal species generated IAA in varied degrees, ranging from 1.95 to 11.16 μg/mL (Fig. 1). The results revealed that the most indole acetic acid IAA productive species were T4 isolated from *Thymus vulgaris* (11.16 μg/mL) and T7 isolated from sweet basil (8.78 μg/mL).

There has been a lot of interest in the interactions between plants and the rhizosphere-associated microbes, like fungus, since being aware of these processes could lead to ecologically friendly farming methods. Roots create a large variety of biological substances, including organic acids, sugars, and vitamins. Then, fungal communities use these as signals or as nutrition [47]. On the other hand, fungi release phytohormones, volatile chemicals, and siderophores, which can either indirectly or directly promote plant development by making their host more nutrient-available [48]. Several endophytic bacteria with valuable metabolites for agriculture and biotechnology are present in different medicinal plants [49, 50].

Regarding the phytohormone-like substances generated from fungal species, filtrate examination of three endophytic fungi isolated from healthy plants revealed that they were able to produce gibberellin-like compounds as well as indole acetic acid [51]. Also, Khalil et al*.* [43] reported that all isolated endophytic fungal strains succeed to produce varying quantities of ammonia and indole acetic acid. This is supported by Shi et al*.* [52] and Vinale et al*.* [53] who revealed that some endophytic bacteria can produce phytohormones such as indole 3-acetic acid. These findings align with ours that suggested varying qualitative IAA production was present from all isolated endophytic fungal strains. The variances in IAA production among endophytic fungi could be ascribed to genetic variables that control productivity [54].

### Screening for the most promising production medium

**Fig. 2:**
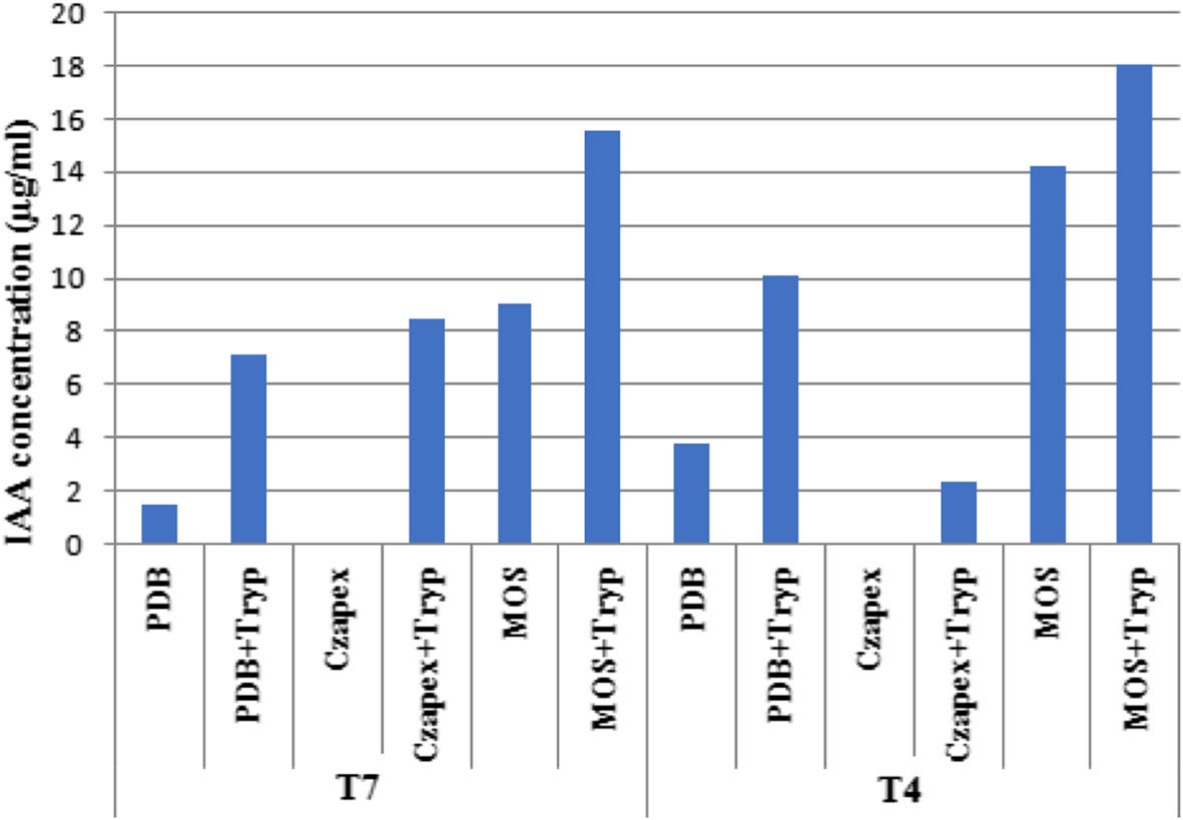
Screening for the most promising production medium for indole acetic acid (IAA) using thyme isolate (T4) and sweet basil isolate (T7)

The fungal media that were used in this experiment were PDB, PDB + Tryp, Czapex, Czapex + Tryp, MOS and MOS + Tryp. The results (Fig. 2) revealed that when screening for the most promising production medium using T4 species and T7 species as the most potent species for IAA production, the fungal species T4 produced a high amount of IAA (18.02 µg/ml) at MOS + Tryp medium and produce low amount (1.45 µg/ml) at PDB medium. While, the fungal species T7 produce high amount of IAA at MOS + Tryp medium (15.58 µg/ml)) and produce low amount at Czapex + Tryp medium (1.45 µg/ml). Also, the results showed that the fungal (T4 and T7) cannot produce IAA at Czapex medium. Estimating the amount of IAA in the culture medium showed that there were differences in the amounts of IAA in the culture extracts. All endophytes had larger concentrations of IAA in media treated with tryptophan than cultures in media lacking tryptophan.

These findings are similar with those of Biswas et al*.* [55] and Fouda et al*.* [56], who discovered varying quantities of IAA in culture extracts and found that broths treated with tryptophan had higher concentrations of IAA for all endophytes when compared to cultures in broth without tryptophan. Similarly, Khalil et al*.* [43] who employed tryptophans a precursor for IAA synthesis, discovered that endophytic fungal species produced higher IAA when tryptophan was present at a concentration of 5 mg/L compared to the absent of tryptophan from Czapek Dox broth. So, several bacteria that promote plant growth have the well-established ability to synthesize IAA [56]. Generally, IAA is synthesized by two different pathways, the Trp-independent and the Trp-dependent, the latter of which is still difficult for most bacteria to use [57], although it has been identified in a few different species of microorganisms [58]. While, bacteria can easily synthesize IAA through four major Trp-dependent pathways [59].

Furthermore, it has been demonstrated that sources of carbon and nitrogen are critical in determining the generation of IAA by bacteria and fungi. Temperature and pH level are just two of the many environmental variables that might affect IAA biosynthesis. According to Strzelczyk et al*.* [60] auxin production is preferred by mycorrhizal fungi at pH 6.0–9.0. Comparable patterns have been noted in the white rot fungus *Pleurotus ostreatus* and the pathogenic fungus *Nectria pterospermi*, which affects the canker of *Pterospermum* with maple leaves. Many researches confirmed that the medium's pH level affects the synthesis of IAA and acidic pH is preferred for fungal production of IAA compared with neutral and alkaline pH [61]. It was proposed that in vitro IAA release is the primary source of pH drop, or that IAA accumulation is directly proportionate to pH decrease, since the pH value of the surroundings directly affects cell growth [61].

#### Macroscopic and microscopic examination

**Fig. 3:**
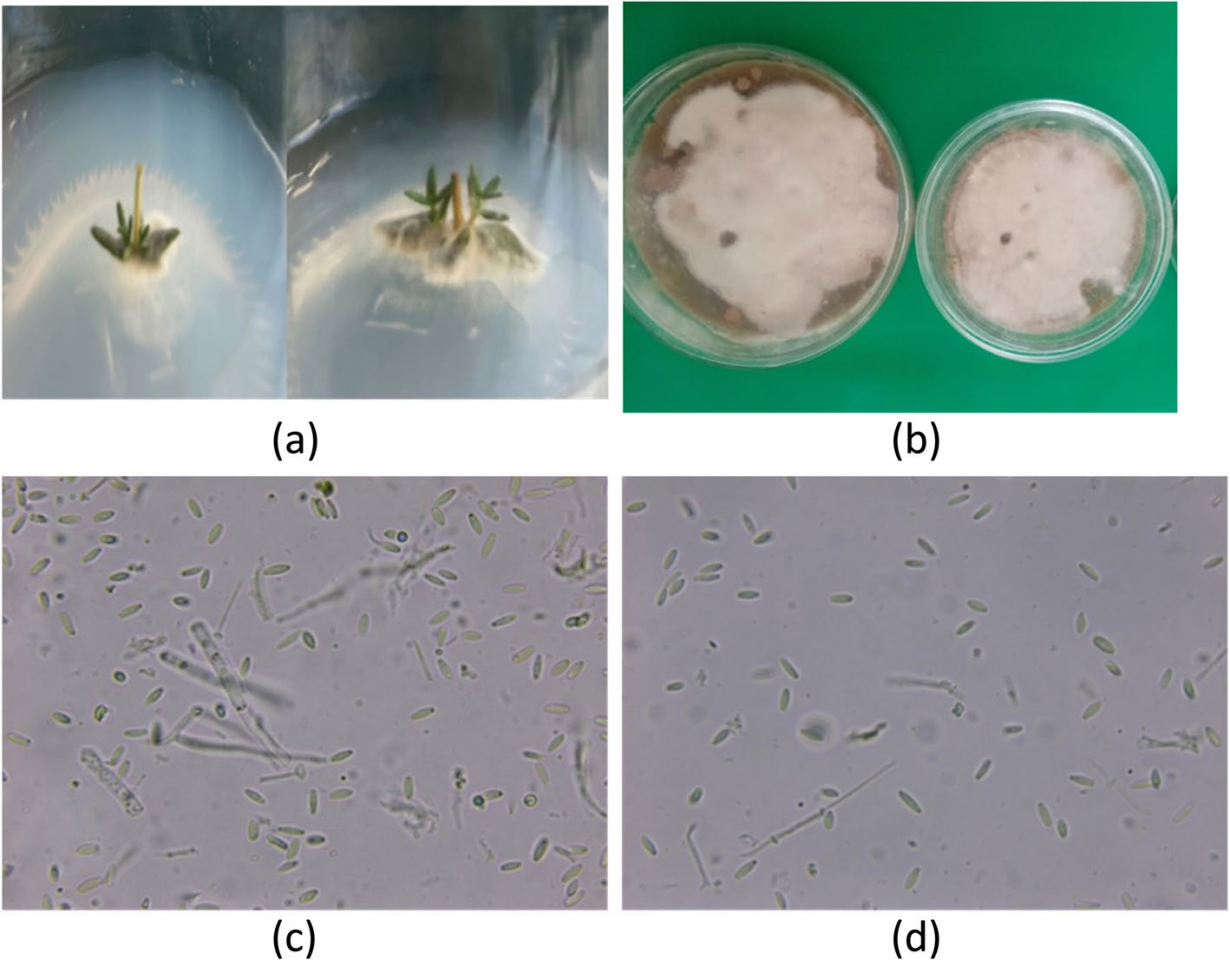
*N. aotearoa* (**a**) fungi growing from sterilized thyme plant sample, (**b**) fungal growth on PDA medium, (**c** and **d**) conidiophore and spores were photographed under (400x) light microscope

Based on the preliminary screening findings, we picked and identified the most active fungal species for identification. The macroscopic, microscopic examination indicated that it may be *Neopestalotiopsis aotearoa*. Figure 3a shows the shape of *N. aotearoa* growth on the sterilized thyme plant sample, and Fig. 3b shows the growth on PDA. The fungal colonies were grown on PDA and produced aerial mycelium characterized by an abundant white cottony mycelium and a dark-purple undersurface. Figures c and d showed the shape of* N. aotearoa* under the light microscope and indicated that microconidia and megacoindia were not found. macroconidia were straight, cylindrical, and curved at both ends; their measurements indicated a size of 44–48 × 3.5–4.3 µm with 1 septate.

### Molecular identification

The sequence analysis 18S rRNA indicated that the strain was *Neopestalotiopsis aotearoa* and given the accession number (OR872336) in NCBI Genbank. According to our search; it is the first record for isolation of *N. aotearoa* endophyte from *Thymus vulgaris*.

### Statistical modeling and optimization of IAA production

**Fig. 4:**
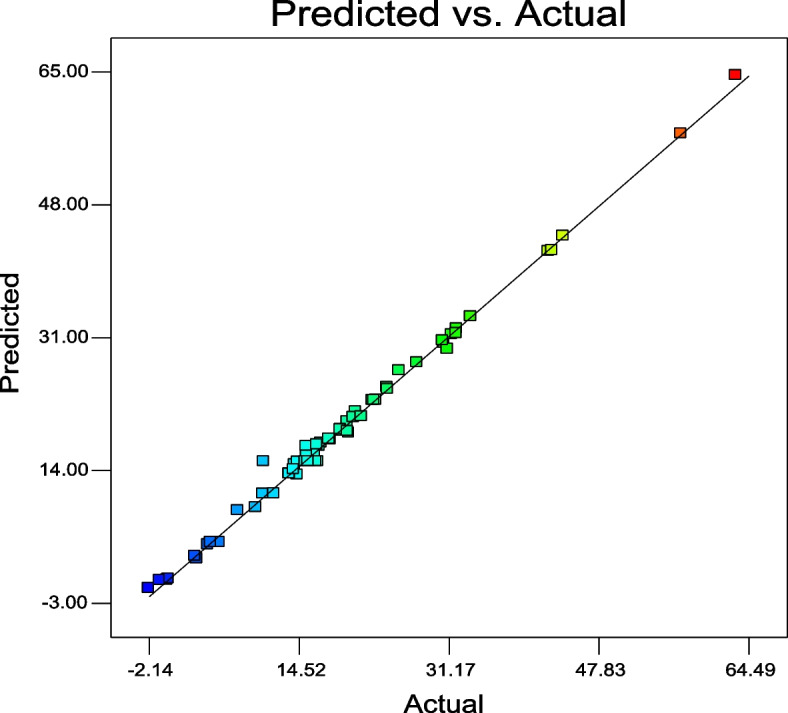
Graphical representation of the correlation between experimental and predicted IAA results from *N. aotearoa*

The design of Box-Behnken resulted in 62 runs that were experimentally tested, and IAA was evaluated at the end of the test of *Neopestalotiopsis aotearoa.* The results of the IAA production are represented in Table 2, reaching 63.13 µg/mL, and the correlation between the experimental and predicted values is graphically represented in Fig. 4.

**Table 2:** IAA production resulted from the Box-Behnken design for *N. aotearoa*

**Run**	**IAA production (µg/ml)**			**Run**	**IAA production (µg/ml)**		
	**Experimental value**	**Predicted value**	**Residual**		**Experimental value**	**Predicted value**	**Residual**
1	16.723	15.073	1.650	**32**	22.720	22.932	-0.212
2	15.511	15.511	0.000	**33**	5.708	4.726	0.982
3	15.602	15.073	0.529	**34**	4.748	4.748	0.000
4	-0.097	-0.097	0.000	**35**	43.936	43.936	0.000
5	10.657	15.073	-4.416	**36**	63.128	64.492	-1.364
6	31.538	31.326	0.212	**37**	20.119	18.755	1.364
7	15.410	17.040	-1.630	**38**	42.684	42.099	0.585
8	3.012	2.964	0.048	**39**	33.681	33.632	0.049
9	-0.933	-0.106	-0.827	**40**	31.082	29.453	1.630
10	16.444	15.073	1.371	**41**	23.164	22.952	0.212
11	24.435	24.329	0.105	**42**	15.271	15.073	0.198
12	16.295	16.295	0.000	**43**	30.547	30.547	0.000
13	16.565	17.239	-0.674	**44**	57.055	57.055	0.000
14	24.374	24.586	-0.212	**45**	19.179	19.179	0.000
15	16.860	17.072	-0.212	**46**	22.930	22.930	0.000
16	32.094	32.094	0.000	**47**	18.052	17.840	0.212
17	21.511	20.837	0.674	**48**	19.225	19.225	0.000
18	9.775	9.191	0.585	**49**	19.970	20.181	-0.212
19	17.033	17.479	-0.446	**50**	14.076	14.661	-0.585
20	7.784	8.792	-1.008	**51**	17.951	17.951	0.000
21	19.204	18.992	0.212	**52**	-2.143	-1.161	-0.982
22	14.383	13.375	1.008	**53**	20.891	21.475	-0.585
23	15.432	16.016	-0.585	**54**	3.228	2.630	0.598
24	13.498	13.498	0.000	**55**	27.696	27.745	-0.049
25	19.979	18.971	1.008	**56**	42.322	41.989	0.333
26	14.444	15.042	-0.598	**57**	15.742	15.073	0.669
27	20.626	20.731	-0.105	**58**	32.055	31.470	0.585
28	13.997	14.045	-0.048	**59**	30.684	30.238	0.446
29	25.714	26.722	-1.008	**60**	10.599	10.932	-0.333
30	4.441	4.441	0.000	**61**	13.526	13.526	0.000
31	11.802	10.975	0.827	**62**	0.064	0.064	0.000

The analysis of variance (ANOVA) results (Table 3) revealed significant model due to the *F*-value of 63.29 and the model terms of C, D, E, F, AB, AC, AE, AF, BE, CE, CF, CG, DE, DF, DG, EF, EG, C^2^, D^2^, E^2^, F^2^, G^2^, ACE, BEG, A^2^B, A^2^C, A^2^D, A^2^E, A^2^F, B^2^C, B^2^E, B^2^G and BC^2^.

**Table 3:** ANOVA of the results obtained from Box-Behnken design

Source	Sum of squares	Df	Mean square	*F*-value	*p*-value Prob > F
**Model**	9764.723	47	207.7601	63.29482	< 0.0001
**A-D( +) Mannose**	10.04543	1	10.04543	3.060375	0.1021
**B-Mannitol**	12.87511	1	12.87511	3.922448	0.0676
**C-Tryptophan**	938.1427	1	938.1427	285.8084	< 0.0001
**D-Potassium Nitrate**	334.8678	1	334.8678	102.0186	< 0.0001
**E-Ammonium chloride**	1710.858	1	1710.858	521.2189	< 0.0001
**F-Temperature**	215.4828	1	215.4828	65.64757	< 0.0001
**G-Initial Ph**	0.075683	1	0.075683	0.023057	0.8815
**AB**	203.4801	1	203.4801	61.9909	< 0.0001
**AC**	159.8189	1	159.8189	48.68939	< 0.0001
**AD**	5.619908	1	5.619908	1.712124	0.2118
**AE**	88.61892	1	88.61892	26.99806	0.0001
**AF**	95.98915	1	95.98915	29.24342	< 0.0001
**AG**	0.755815	1	0.755815	0.230262	0.6387
**BC**	0.08925	1	0.08925	0.02719	0.8714
**BD**	13.24013	1	13.24013	4.03365	0.0643
**BE**	54.93368	1	54.93368	16.73573	0.0011
**BG**	0.008741	1	0.008741	0.002663	0.9596
**CE**	465.8205	1	465.8205	141.9138	< 0.0001
**CF**	27.99976	1	27.99976	8.530223	0.0112
**CG**	105.0589	1	105.0589	32.00656	< 0.0001
**DE**	125.5318	1	125.5318	38.24369	< 0.0001
**DF**	56.61903	1	56.61903	17.24918	0.0010
**DG**	21.29327	1	21.29327	6.487067	0.0233
**EF**	193.7278	1	193.7278	59.01983	< 0.0001
**EG**	192.086	1	192.086	58.51965	< 0.0001
**FG**	15.07489	1	15.07489	4.592617	0.0502
**A**^**2**^	1.836756	1	1.836756	0.559574	0.4668
**B**^**2**^	2.909283	1	2.909283	0.886323	0.3624
**C**^**2**^	405.3527	1	405.3527	123.4921	< 0.0001
**D**^**2**^	217.2602	1	217.2602	66.18907	< 0.0001
**E**^**2**^	527.0339	1	527.0339	160.5627	< 0.0001
**F**^**2**^	304.3096	1	304.3096	92.70896	< 0.0001
**G**^**2**^	86.63827	1	86.63827	26.39465	0.0002
**ACE**	86.68723	1	86.68723	26.40956	0.0002
**AFG**	8.140429	1	8.140429	2.48001	0.1376
**BEG**	31.96354	1	31.96354	9.737802	0.0075
**A**^**2**^**B**	157.6399	1	157.6399	48.02553	< 0.0001
**A**^**2**^**C**	22.18152	1	22.18152	6.757678	0.0210
**A**^**2**^**D**	37.434	1	37.434	11.4044	0.0045
**A**^**2**^**E**	202.2188	1	202.2188	61.60665	< 0.0001
**A**^**2**^**F**	40.98233	1	40.98233	12.48541	0.0033
**A**^**2**^**G**	7.849364	1	7.849364	2.391336	0.1443
**AC**^**2**^	10.03602	1	10.03602	3.057509	0.1022
**B**^**2**^**C**	61.51994	1	61.51994	18.74226	0.0007
**B**^**2**^**E**	426.2217	1	426.2217	129.8499	< 0.0001
**B**^**2**^**G**	113.4808	1	113.4808	34.57231	< 0.0001
**BC**^**2**^	33.85365	1	33.85365	10.31363	0.0063
**Residual**	45.95385	14	3.282418		
**Lack of fit**	21.0801	9	2.342233	0.470824	0.8461
**Pure error**	24.87375	5	4.974751		
**Cor total**	9810.677	61			

Where, A: D ( +) Mannose, B: Mannitol, C: Tryptophan, D: Potassium nitrate, E: Ammonium chloride, F: Temperature and G: Initial pH. The model "Lack of Fit *F*-value" was not significant (0.47) which reflects the fitting of the data to the model. This fitting was confirmed by the R^2^ of (0.99), adjusted-R^2^ of (0.98) and signal adequate precision of (41.18). All data analysis showed that the produced model can be used to navigate the data in the model space.

**Fig. 5:**
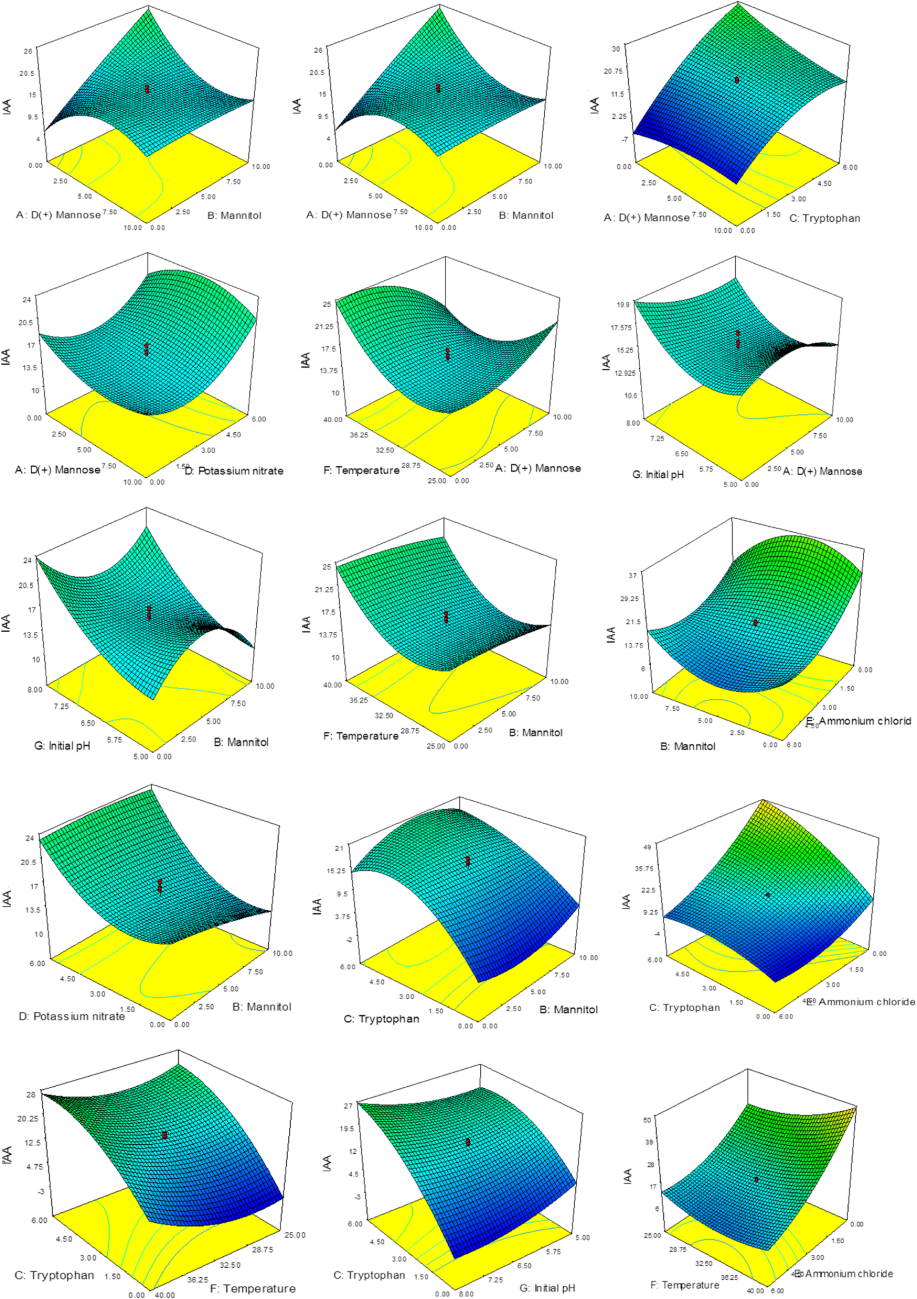
3D surface display of factor-factor interactions and their effect on IAA production

The produced model can be represented in the following equation and factor-factor interactions are shown in Fig. 5:

$$\mathbf{IAA}\boldsymbol\;\boldsymbol(\boldsymbol\mu\mathbf g\boldsymbol/\mathbf{ml}\boldsymbol)=\;75.38\;+3.78\;\ast\;\mathrm A\;+15.53\ast\;\mathrm B\;+\;10.33\;\ast\;\mathrm C\;-\;2.91\;\ast\;\mathrm D\;+\;16.82\;\ast\;\mathrm E\;-5.73\;\ast\;\mathrm F\;-\;13.47\;\ast\;\mathrm G\;-\;0.70\;\ast\;\mathrm A\;\ast\;\mathrm B\;-0.65\;\ast\;\mathrm A\;\ast\;\mathrm C\;+\;0.41\;\ast\;\mathrm A\;\ast\;\mathrm D\;-0.95\;\ast\;\mathrm A\;\ast\;\mathrm E\;+\;0.17\;\ast\;\mathrm A\;\ast\;\mathrm F\;+\;0.17\;\ast\;\mathrm A\;\ast\;\mathrm G\;+\;0.14\;\ast\;\mathrm B\;\ast\;\mathrm C\;+\;0.09\;\ast\;\mathrm B\;\ast\;\mathrm D\;-\;1.78\ast\;\mathrm B\;\ast\;\mathrm E\;-\;1.69\;\ast\;\mathrm B\;\ast\;\mathrm G\;-\;1.21\ast\;\mathrm C\;\ast\;\mathrm E\;-\;0.08\;\ast\;\mathrm C\;\ast\;\mathrm F\;+\;0.81\;\ast\;\mathrm C\;\ast\;\mathrm G\;-\;0.44\;\ast\;\mathrm D\;\ast\;\mathrm E\;+\;0.12\;\ast\;\mathrm D\;\ast\;\mathrm F\;-\;0.36\;\ast\;\mathrm D\;\ast\;\mathrm G\;-\;0.22\;\ast\;\mathrm E\;\ast\;\mathrm F\;-\;1.53\;\ast\;\mathrm E\;\ast\;\mathrm G\;+\;0.21\ast\;\mathrm F\;\ast\;\mathrm G\;-\;0.30\;\ast\;\mathrm A^2\;-\;1.19\;\ast\;\mathrm B^2\;-\;0.78\;\ast\;\mathrm C^2\;+\;0.45\ast\;\mathrm D^2\;+\;0.69\;\ast\;\mathrm E^2\;+\;0.08\;\ast\;\mathrm F^2\;+\;1.12\;\ast\;\mathrm G^2\;+\;0.07\;\ast\;\mathrm A\;\ast\;\mathrm C\;\ast\;\mathrm E\;-\;0.02\;\ast\;\mathrm A\;\ast\;\mathrm F\;\ast\;\mathrm G\;+\;0.09\;\ast\;\mathrm B\;\ast\;\mathrm E\;\ast\;\mathrm G\;+\;0.05\;\ast\;\mathrm A^2\;\ast\;\mathrm B\;+\;0.03\;\ast\;\mathrm A^2\;\ast\;\mathrm C\;-\;0.04\;\ast\;\mathrm A^2\;\ast\;\mathrm D\;+\;0.09\ast\;\mathrm A^2\;\ast\;\mathrm E\;-\;0.01\;\ast\;\mathrm A^2\;\ast\;\mathrm F\;+\;0.03\;\ast\;\mathrm A^2\;\ast\;\mathrm G\;-\;0.03\;\ast\;\mathrm A\;\ast\;\mathrm C^2\;-\;0.05\;\ast\;\mathrm B^2\;\ast\;\mathrm C\;+\;0.14\;\ast\;\mathrm B^2\;\ast\;\mathrm E\;+\;0.14\;\ast\;\mathrm B^2\;\ast\;\mathrm G\;+\;0.06\;\ast\;\mathrm B\;\ast\;\mathrm C^2$$

Where, A: D ( +) Mannose; B: Mannitol; C: Tryptophan; C: Potassium nitrate; E: Ammonium chloride; F: Temperature; G: Initial pH.

According to the produced model maximum IAA productivity (63.40 µg/ml) can be reached under the following conditions: 5.52 g/l, D ( +) Mannose; 6.60 g/l, Mannitol; 5.94 g/l, Tryptophan; 1.80 g/l, Potassium nitrate at pH of 7.84 and incubation temperature of 39.80°C.

### Germination experiment

#### Effects of IAA produced by *N. aotearoa* on seed germination and root growth

It was reported that IAA generated by fungi has the ability to promote the growth of root hair and lateral roots [62]. The plants that are related with it absorb nutrients more effectively because of the stimulation of root growth and development. Thus, the biomass production of shoots or/and fruits has been increased [63].

**Fig. 6:**
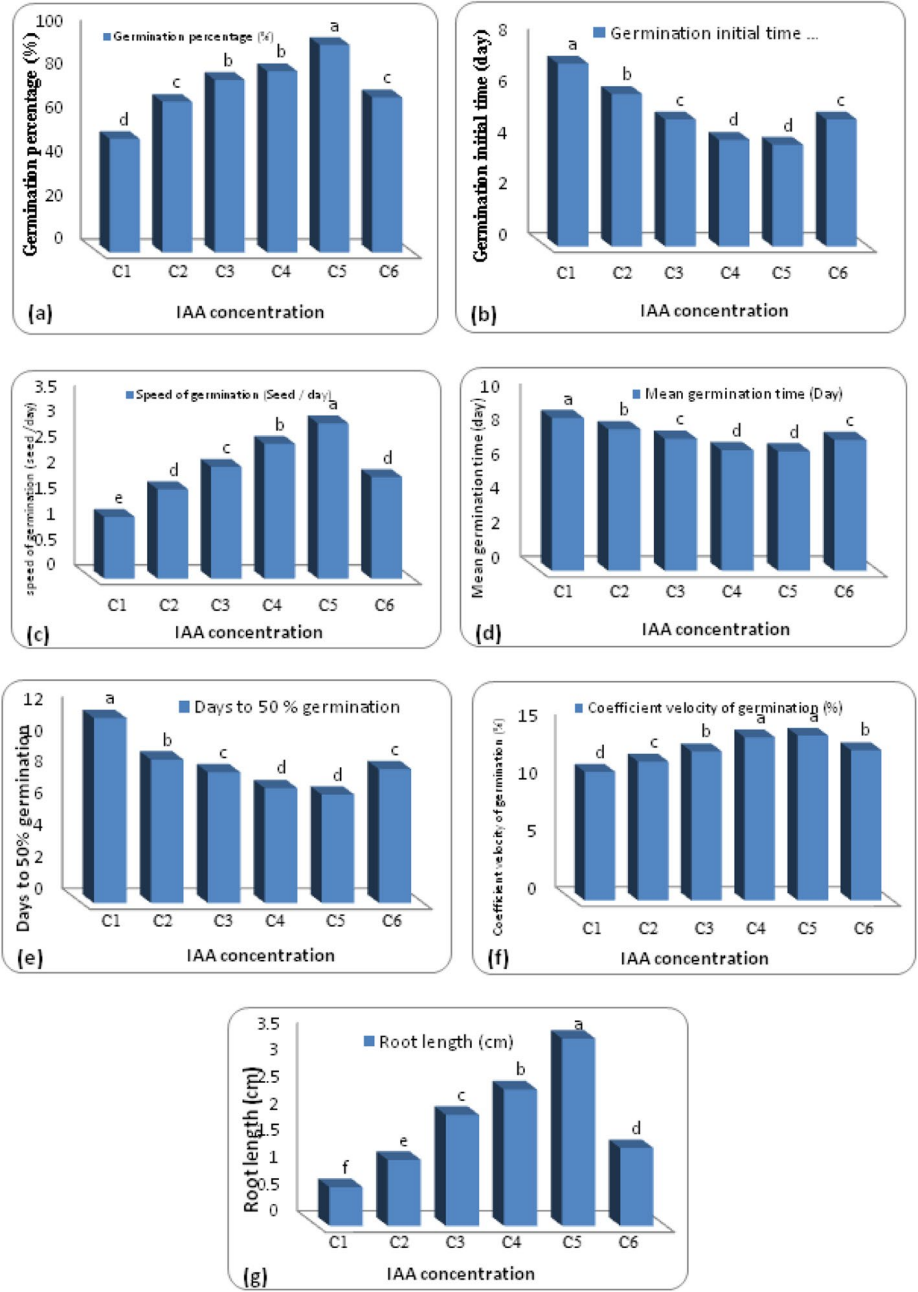
Germination percentage (**a**), Germination initial time (**b**), speed of germination (**c**), Mean germination time (**d**), Days to 50% germination (**e**), Coefficient velocity of germination (**f**) and Root length (**g**) of sweet basil seeds under different concentrations of extracted IAA. C1, 0 ppm; C2, 25 ppm; C3, 50 ppm; C4, 75 ppm; C5, 100 ppm and C6, 150 ppm. The same letter at the top of the columns means they are not significantly different at the *P* < 0.05 level of significance

**Fig. 7:**
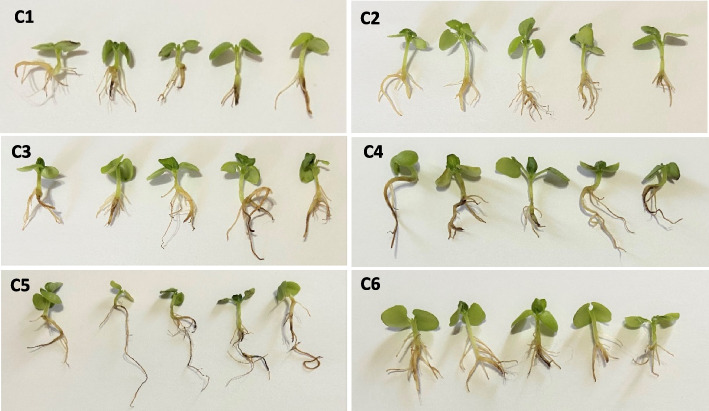
Effect of different extracted IAA concentrations on germination parameters of sweet basil (*O. basilicum*). C1, 0 ppm; C2, 25 ppm; C3, 50 ppm; C4, 75 ppm; C5, 100 ppm and C6, 150 ppm

In the current study, the characters of germinated seedling of sweet basil in extracted IAA concentrations and control throughout the 14 days of germination were investigated (Table 4 and Fig. 6). Highly significant differences were shown by analysis of variance between all IAA concentrations and control for all germination parameters, with superiority to the rate of C5 ppm, which had the highest value of germination percentage (95 ± 1.6), speed of germination (3.04 ± 0.05), and root length (3.49 ± 0.07) with significant differences as compared with other IAA rates (Figs. 6a-c-g and 7). Moreover, treating seeds with IAA at C5 and C4 ppm exhibited higher coefficient velocity of germination (14.41 ± 0.19 and 14.26 ± 0.23, respectively) with insignificant difference between values (Fig. 6f).

**Table 4:** Effect of different extracted IAA concentrations on the seed germination parameters of sweet basil (*O. basilicum*)

IAA concentration (ppm)	Germination percentage (%)	Germination initial time (day)	speed of germination (seed /day)	Mean germination time (day)	Days to 50% germination	Coefficient velocity of germination (%)	Root length (cm)
**0**	52 ± 2.0d	7.2 ± 0.2a	1.21 ± 0.06e	8.89 ± 0.08a	11.6 ± 0.24a	11.24 ± 0.10d	0.72 ± 0.02f
**25**	69 ± 1.0c	6.0 ± 0.0b	1.75 ± 0.02d	8.24 ± 0.09b	9.0 ± 0.0b	12.14 ± 0.13c	1.23 ± 0.10e
**50**	79 ± 4.0b	5.0 ± 0.0c	2.19 ± 0.14c	7.68 ± 0.15c	8.2 ± 0.20c	13.04 ± 0.27b	2.07 ± 0.06c
**75**	83 ± 2.5b	4.2 ± 0.2d	2.64 ± 0.13b	7.02 ± 0.11d	7.2 ± 0.20d	14.26 ± 0.23a	2.54 ± 0.07b
**100**	95 ± 1.6a	4.0 ± 0.0d	3.04 ± 0.05a	6.95 ± 0.09d	6.8 ± 0.20d	14.41 ± 0.19a	3.49 ± 0.07a
**150**	71 ± 1.9c	5.0 ± 0.0c	1.99 ± 0.04cd	7.61 ± 0.07c	8.4 ± 0.24c	13.14 ± 0.13b	1.45 ± 0.05d

Mean followed by the same letter within columns are not significantly different at the *P* < 0.05 level of significance using Duncan’s multiple range test. Values reported are the means ± standard error *(n* = *6*)

On the contrary, the lowest values of germination initial time (4.0 days), mean germination time (6.95 ± 0.09 days), and days to 50% germination (6.80 ± 0.20 days) were obtained with IAA at C5 ppm, while the highest values (7.20 ± 0.2, 8.89 ± 0.08, 11.60 ± 0.24 days, respectively) were obtained in the control treatment. Also, the data presented in Table 4 indicated that raising the IAA concentration to a high dose (C6 ppm) led to a negative impact and a significant decrease in all germination parameters (Figs. 6 and 7).

In this investigation, all extracted IAA treatments significantly improved germination and seedling growth of sweet basil, when compared with the control seedlings. The growth-promoting impact of exogenous IAA on seed germination might be attributed to their indirect influence as Zhao et al. [24] demonstrated that IAA priming may enhance seed germination by modifying the production and balance of endogenous phytohormones in ways that are compatible with what is known about these hormones from the literature, i.e., increasing the GA and IAA contents and decreasing the ABA contents. This evidence is backed by the fact that GA and IAA positively accelerate plant development whereas ABA negatively inhibits plant growth [64–66].

In addition to its mediating role in the synthesis of endogenous phytohormones, IAA priming may stimulate seedling development by modulating the sucrose metabolism pathway and protein content in germinated seedlings. Zhao et al*.* [24] proved that the activities of enzymes linked to sucrose and the amounts of soluble sugar were markedly elevated, which sped up the growth of seedlings. As carbohydrate have pivotal functions that support growth and a signal to control gene expression and interact with other signaling pathways, such as phytohormone signaling so sucrose is a vital component of development [67]. Besides, it was reported that sweet basil, coriander, and peppermint seedlings that germinated had higher total protein contents as a response of IAA treatment leading to enhance the seed germination as compared with control [35]. Additionally, IAA can influence enzymes activity, such as glyoxalase I, which in germinating pea seeds was regulated by IAA and resulted in higher rates of cell proliferation and development [68].

In this study, it was showed that the application of high IAA concentration had inhibition effect on seed germination and root length. These findings are consistent with those obtained by Nakamura et al. [26] showed that the ideal concentration of IAA stimulated seed germination whereas a larger quantity prevented it. Additionally, it appears that the hormones tested had a higher inhibitory effect on seedling root development than germination percentage [69]. Concerning the suppressive impact of high IAA application, Shuai et al. [70] suggested that IAA can prevent seeds from germinating by increasing the ABA biosynthesis pathway and negatively mediating GA biosynthesis. The paradoxical impact of IAA administration might be attributable to species diversity or applied concentration [70]. The use of IAA not only enhances plant growth and germination, but enhances grain quality, carbohydrate and protein content [61].

## Conclusions

The present study showed that medicinal plants are a rich ecological source for diverse endophytic fungi. Several endophytes were isolated from different ornamental and medicinal plants (i.e., *V. major, B. disticha, D. plumieri, T. vulgaris, S. officinalis, R. officinalis, and O. basilicum*) and exhibited a variable capacity in vitro for effective activities in the production of phytohormones like indole 3-acetic acid (IAA). The selected species; *Neopestalotiopsis aotearoa*, recorded for the first time and was the most effective species for IAA production. The produced IAA implied a vital role in the improvement of sweet basil (*O. basilicum*) growth, so it could be used as a natural bio-stimulant in agriculture to improve plant growth and decrease the risk of using synthetic chemicals in the environment.

### Supplementary Information

Supplementary Material 1.

## Data Availability

Data of Gene Bank NCBI for fungal identification is available at: https://blast.ncbi.nlm.nih.gov/Blast.cgi.
