# Supplementary material for: Optimisation of indole acetic acid production by Neopestalotiopsis aotearoa endophyte isolated from Thymus vulgaris and its impact on seed germination of Ocimum basilicum
Source: BMC Biotechnol. 2024 Jul 6;24:46. doi: 10.1186/s12896-024-00872-3 (PMC11227711; doi:10.1186/s12896-024-00872-3)
Supplement: Supplementary file 1 — Supplementary Material 1. [file 12896_2024_872_MOESM1_ESM.docx]

**Table (S1):** Screening for the most potent IAA-producing isolate on (PDB + Tryp) medium.

| **Isolate code** | **Isolation plant source** | **IAA (**µ**g/ml)** |
| --- | --- | --- |
| T1 | *Vinca major* | 1.946 |
| T2 | *Breynia disticha* | 3.778 |
| T3 | *Duranta plumieri* | 2.790 |
| T4 | *Thymus vulgaris* | 11.164 |
| T5 | *Salvia officinalis* | 3.854 |
| T6 | *Rosmarinus officinalis* | 5.206 |
| T7 | *Ocimum basilicum* | 8.784 |

**Table (S2):** Screening for the most promising production medium for indole acetic acid (IAA) using thyme isolate (T4) and sweet basil isolate (T7).

| **Isolate** | **Medium** | **IAA (µg/ml)** |
| --- | --- | --- |
| **T4** | PDB | 3.740 |
|  | PDB+Tryp | 10.129 |
|  | Czapex | 0 |
|  | Czapex+Tryp | 2.325 |
|  | MOS | 14.244 |
|  | MOS+Tryp | 18.015 |
| **T7** | PDB | 1.446 |
|  | PDB+Tryp | 7.138 |
|  | Czapex | 0 |
|  | Czapex+Tryp | 8.483 |
|  | MOS | 9.018 |
|  | MOS+Tryp | 15.575 |
